# Vitamin D neuroimmune synaptic plasticity and precision intervention in pediatric neurodevelopmental disorders

**DOI:** 10.3389/fnagi.2026.1895712

**Published:** 2026-08-05

**Authors:** Cheng Xie, Ping Xiang, Xinyun Ye, Wei Zhang, Yue Qian, Ying-ying Mu, Qiuhua Jiang

**Affiliations:** 1 Department of Neurosurgery, Ganzhou Hospital-Nanfang Hospital, Southern Medical University (Ganzhou People’s Hospital), Ganzhou, China; 2 Department of Pathology, Zunyi Hospital of Traditional Chinese Medicine, Zunyi, Guizhou, China; 3 Medical Affairs Department, The Second Affiliated Hospital of Zunyi Medical University, Zunyi, Guizhou, China; 4 Department of Pathogen Biology, Guizhou Nursing Vocational College, Guiyang, Guizhou, China

**Keywords:** attention-deficit/hyperactivity disorder, autism spectrum disorder, children, epilepsy, individualized intervention, neuroimmune–synaptic plasticity axis, neurological disorders, vitamin D

## Abstract

Vitamin D deficiency is highly prevalent in children worldwide but has long been regarded only as a risk factor for bone health. Emerging evidence indicates that vitamin D profoundly influences child brain development by regulating neuroimmune responses and synaptic plasticity, forming a “neuroimmune–synaptic plasticity axis.” Deficiency is significantly associated with the risk of multiple pediatric neurological disorders, including autism spectrum disorder (ASD), epilepsy, and attention-deficit / hyperactivity disorder (ADHD). This axis exerts irreversible regulatory effects during critical developmental windows from gestation to the preschool period, representing a core causal pathway linking vitamin D deficiency to neurodevelopmental disorders. However, the clinical benefits of vitamin D supplementation are highly heterogeneous, necessitating a move beyond the “one-size-fits-all” paradigm. This review systematically integrates causal mechanisms, molecular pathways, disease-specific associations, and clinical evidence, proposing the “neuroimmune–synaptic plasticity axis” as a central hypothesis. We critically analyze the mechanisms of vitamin D during key neurodevelopmental periods and evaluate sources of heterogeneity in existing clinical trials, including dosage, timing, VDR genotype, and co-nutrients. Finally, we propose a research roadmap for individualized supplementation strategies based on developmental stage, biomarkers, and VDR genotype, aiming to transition vitamin D use from empirical to precision intervention.

## 1 Introduction

Vitamin D, an essential fat-soluble vitamin, has traditionally been recognized for its role in calcium-phosphate metabolism and bone health (Delrue and Speeckaert, 2023a). However, recent studies have revealed its critical importance in the development and function of the nervous system, particularly during childhood (Cianferotti et al., 2017; Joshi and Uday, 2023; Ortiz-Prado et al., 2025). An unresolved core question is whether vitamin D deficiency acts as a plausible contributing risk factor for pediatric neurological disorders or merely an epiphenomenon; most existing human observational data only support correlational relationships rather than definitive causality. To address this, we propose the “neuroimmune–synaptic plasticity axis” as the scientific hypothesis throughout this review: vitamin D coordinates microglia-mediated neuroinflammation (Brustad and Meyer, 2014), synaptic pruning (Haddad et al., 1993), and excitatory/inhibitory (E/I) balance (Kouba et al., 2023) to influence child brain development and disease susceptibility. This axis dynamically operates during three critical windows – mid-early pregnancy, the postnatal synaptic burst period (0–2 years), and the preschool prefrontal myelination period – and its disruption may contribute to the emergence of irreversible neural circuit abnormalities observed in preclinical animal models (Holick, 2017). Maternal vitamin D status during pregnancy affects fetal neurodevelopment via the placenta, which is potentially relevant to early childhood cognition, language, motor skills, and social-emotional development. Globally, vitamin D deficiency is a prevalent health problem, affecting nearly half of the population, especially pregnant women (Holick, 2012).

Childhood is a critical period of rapid nervous system development, and vitamin D deficiency may exert profound correlational effects on neural network formation and brain function. Beyond regulating calcium balance, vitamin D participates in central nervous system development and maintenance by modulating neurotransmitters, neuroprotection, and neuroinflammation (Ajoolabady et al., 2025; Tuohimaa et al., 2009). Furthermore, vitamin D regulates neurodevelopment-related metabolic pathways such as tryptophan and fatty acid metabolism (Savran et al., 2025), influencing phenotypic manifestations in children such as communication delays and respiratory dysfunction, suggesting therapeutic potential in neurodevelopmental disorders like ASD (Christakos et al., 2016).

Clinical observations also link vitamin D deficiency to multiple pediatric neurological diseases. For example, children with chronic neurological conditions [including cerebral palsy (Alenazi and Alanezi, 2024), epilepsy (Sourbron et al., 2024), and neurodegenerative diseases (Plantone et al., 2022)] commonly have low vitamin D levels, which are associated with motor impairment, cognitive decline, and developmental delay (Isik et al., 2023; Moretti et al., 2018). Vitamin D deficiency is also associated with impaired executive function in pediatric epilepsy patients, suggesting a protective role in neurocognitive function (Xu et al., 2019). Among children with ASD, vitamin D deficiency is highly prevalent and may act as a risk factor for ASD onset and progression (Alzghoul, 2019; Mazahery et al., 2016; Vinkhuyzen et al., 2017; Wang et al., 2020).

Although numerous observational studies have revealed associations between vitamin D and pediatric neurological disorders, randomized controlled trials (RCTs) evaluating the benefits of high-dose prenatal vitamin D supplementation on child neurodevelopment have shown inconsistent results. For instance, the Danish COPSAC-2010 cohort study found that high-dose prenatal vitamin D did not significantly improve cognitive, motor, or language development at 2.5 years of age and even showed negative effects on some outcomes (Brustad et al., 2021; Sass et al., 2020). Current research in this field still faces three prominent unresolved gaps: First, there lacks an integrated mechanistic framework to systematically interpret the shared pathological connections between vitamin D deficiency and ASD, epilepsy, ADHD; Second, most existing supplementation schemes adopt uniform universal dosages without stratified precision guidance matched to developmental stages, VDR genotypes and clinical biomarkers; Third, few translational studies construct operable research roadmaps to transform basic molecular mechanisms into individualized clinical intervention regimens for children. Against this background, the present review innovatively establishes the “neuroimmune–synaptic plasticity axis” core hypothesis, comprehensively synthesizes stratified preclinical and human clinical evidence, and proposes a multi-dimensional translational roadmap for precision supplementation, so as to fill the above research vacancies and provide standardized clinical references for pediatric neurological disease management.

## 2 Biological functions of vitamin D and its mechanisms in the nervous system

### 2.1 Vitamin D metabolism and receptor distribution

Vitamin D can be synthesized in the skin upon ultraviolet exposure or obtained from diet, existing mainly as vitamin D2 (ergocalciferol) or D3 (cholecalciferol) (De Luca, 1985; Wasserman et al., 1984). In the body, vitamin D is first hydroxylated in the liver by 25-hydroxylase (mainly CYP2R1) to 25-hydroxyvitamin D [25(OH)D], the major circulating form and indicator of vitamin D status (Pilkey et al., 2023; Zhang et al., 2006). Subsequently, 25(OH)D is converted in the kidney by 1α−hydroxylase (CYP27B1) to the active form 1,25-dihydroxyvitamin D [1,25(OH)2D], which binds to the vitamin D receptor (VDR) to exert biological effects (Norman et al., 1982). The metabolism of 25(OH)D and 1,25(OH)2D is also regulated by the bone-derived hormone fibroblast growth factor 23 (FGF23), forming a complex feedback mechanism (Alonso et al., 2023).

Vitamin D receptor is a nuclear receptor widely distributed in the central nervous system, including the hippocampus, thalamus, and cortex – regions involved in learning, memory, and emotional regulation (Sun and Zhang, 2022). Notably, VDR expression in the child brain shows developmental dynamics, peaking during the late fetal and early postnatal synaptic formation period, indicating an irreplaceable role for vitamin D during this window (Li L. et al., 2022). Moreover, neurons and glial cells in the CNS locally express vitamin D-synthesizing enzymes (e.g., CYP27B1), allowing local synthesis and metabolism, and autocrine/paracrine functions (Delrue and Speeckaert, 2023b; Nikolac Gabaj et al., 2020). This local metabolism enables dynamic regulation of vitamin D activity within the nervous system independently of peripheral supply.

The regulation of vitamin D metabolism and VDR distribution involves multi-level gene expression control. VDR expression is influenced by genetic and environmental factors, and certain VDR polymorphisms (e.g., FokI, BsmI, ApaI) are associated with susceptibility to neurological diseases (Usategui-Martín et al., 2022). Additionally, expression of vitamin D metabolic enzymes is regulated by hormones and signaling pathways to ensure adequate supply under different physiological conditions (De Luca, 1976a,b; Vanherwegen et al., 2017). Overall, the synthesis, metabolism, and receptor distribution of vitamin D constitute a fine-tuned network critical for child nervous system development and function.

### 2.2 Vitamin D in neurodevelopment regulation

Vitamin D, through its active form 1,25(OH)2D binding to VDR, mediates multiple molecular mechanisms that promote neuronal differentiation, axonal growth, and synapse formation. Experimental studies show that vitamin D regulates neuronal gene expression, increasing neurotrophic factors such as brain-derived neurotrophic factor (BDNF; Yurekli and Tunc, 2022) and nerve growth factor (Gezen-Ak et al., 2014), thereby facilitating neural network establishment and function. Vitamin D also regulates the synthesis of neurotransmitters including dopamine, glutamate, and γ−aminobutyric acid (GABA), affecting signal transmission and synaptic plasticity (Gulsen et al., 2024; Kang et al., 2024). Importantly, vitamin D promotes maturation of GABAergic interneurons, and dysregulation of the GABAergic system is a shared pathological basis for ASD and epilepsy.

Vitamin D also plays a significant role in glial cell development. It modulates the functions of astrocytes and microglia, regulating inflammatory responses and supporting neuronal metabolic demands, thereby influencing the neural microenvironment and homeostasis (Dewi et al., 2025). Changes in glial function directly affect neural network construction and repair capacity, and vitamin D’s regulatory role is particularly critical for child brain development (Pertile et al., 2018).

Vitamin D is also involved in regulating key genes for neurodevelopment. For example, it influences neural stem cell proliferation and differentiation, impacting the developmental fate of neurons and glia (Sangha et al., 2023; Uthaiah et al., 2022). It regulates neurotransmitter synthesizing enzymes (e.g., tyrosine hydroxylase), affecting neurotransmitter biosynthesis and neural transmission (Cui et al., 2015). Furthermore, vitamin D may modulate epigenetic mechanisms such as DNA methylation and histone modifications, thereby long-term expression of neurodevelopment-related genes (Bischoff-Ferrari et al., 2025).

In summary, vitamin D shapes child neurodevelopment through at least three parallel pathways: ➀directly regulating neuronal differentiation and synapse formation (dependent on BDNF and the GABAergic system); ➁controlling glial maturation and glia-mediated synaptic pruning; and ➂maintaining ion homeostasis and transmitter synthesis required for E/I balance. These mechanisms collectively form the molecular basis for vitamin D as a “neurodevelopmental modifier” (Supplementary Table 1).

### 2.3 Neuroprotective and immunomodulatory functions of vitamin D

Vitamin D exerts important neuroprotective effects mainly through anti-inflammatory mechanisms that reduce neuroinflammation and protect neurons from damage. Vitamin D suppresses the expression of pro-inflammatory cytokines such as tumor necrosis factor−α (TNF−α; Yu et al., 2025), interleukin-6 (IL-6; Kaviani et al., 2022), and interferon−γ (IFN-γ; Freeley et al., 2018), alleviating neuroinflammation and maintaining CNS homeostasis. Additionally, vitamin D promotes the production of anti-inflammatory cytokines such as IL-10, balancing immune responses in the CNS and avoiding excessive inflammatory damage (Kew, 2019).

Vitamin D modulates the activity of CNS immune cells, particularly microglia and astrocytes, inhibiting their overactivation and preventing neurodegenerative progression (Liu et al., 2025b; Menéndez and Manucha, 2024). Microglia-mediated synaptic pruning is a key process for fine-tuning neural networks during child brain development; vitamin D deficiency can lead to aberrant complement C1q/C3 signaling, causing excessive or insufficient synaptic pruning, thereby contributing to the pathogenesis of ASD and epilepsy. Vitamin D also enhances regulatory T cell (Treg) function, suppresses pro-inflammatory Th1 and Th17 responses, maintains immune tolerance, and reduces the risk of autoimmune neurological diseases (Liu et al., 2025a).

In terms of oxidative stress defense, vitamin D induces antioxidant enzyme expression, reduces free radical generation, and alleviates oxidative damage, thereby protecting neural cells (Kim et al., 2020; Yadav et al., 2019). Vitamin D activates the nuclear factor erythroid 2-related factor 2 (Nrf2) signaling pathway, promoting antioxidant responses and improving neuronal survival (Wang et al., 2023). These neuroprotective mechanisms hold potential for preventing and alleviating various neurological diseases such as multiple sclerosis, Parkinson’s disease, and Alzheimer’s disease (Liu et al., 2026, 2024; Zhang et al., 2024a). Moreover, by maintaining blood-brain barrier integrity and regulating neuronal calcium homeostasis, vitamin D reduces the invasion of harmful substances and inflammatory cells and prevents calcium overload-induced apoptosis, further protecting the nervous system (Zhou et al., 2021).

**FIGURE 1:**
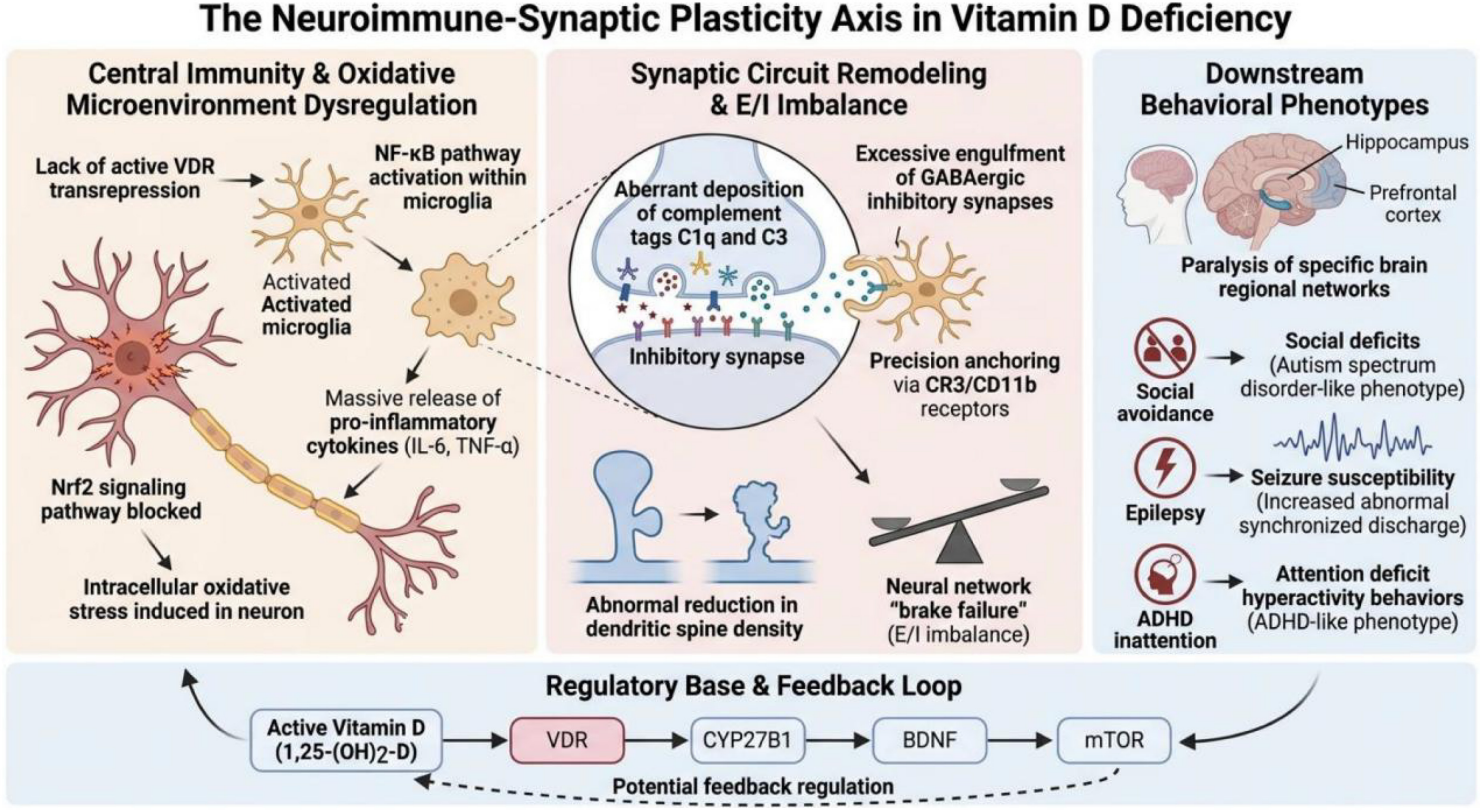
The neuroimmune-synaptic plasticity axis in vitamin D deficiency.

In summary, vitamin D exerts neuroprotective effects in pediatric neurological diseases through anti-inflammatory, immunomodulatory, and antioxidant mechanisms, especially by regulating microglia-mediated synaptic plasticity (Figure 1).

### 2.4 Specific role of vitamin D during critical child brain development windows

Unlike adults, children have multiple critical developmental windows (e.g., late fetal to 2 years: synaptic burst; preschool period: prefrontal myelination). During these windows, vitamin D deficiency may cause irreversible structural and functional changes. Animal experiments show that gestational vitamin D deficiency leads to a >30% reduction in hippocampal GABAergic synapses in offspring, which cannot be fully reversed by supplementation after puberty (Zhang et al., 2024b). Additionally, vitamin D influences neural precursor cell proliferation and differentiation via the Wnt/β−catenin pathway, which is more active during childhood (García-Martínez et al., 2024; González-Sancho et al., 2020; Zou et al., 2024). Therefore, future research should establish dynamic models of vitamin D requirements for different developmental stages rather than applying uniform supplementation regimens.

## 3 Association studies between vitamin D deficiency and pediatric neurological disorders

### 3.1 Autism spectrum disorder and vitamin D

Autism spectrum disorder is a complex neurodevelopmental disorder characterized by social communication deficits, language difficulties, and repetitive stereotyped behaviors; its pathogenesis remains unclear. Epidemiological studies widely report low vitamin D levels in children with ASD and their mothers during pregnancy (Avram et al., 2025). Multiple studies show that serum 25(OH)D levels in children with ASD are significantly lower than in healthy controls, with a higher deficiency rate (Li L. et al., 2022; Wang et al., 2016). Moreover, maternal vitamin D deficiency during pregnancy is significantly associated with increased ASD risk in offspring, especially when deficiency occurs in the first trimester (Hyppönen, 2011; Principi et al., 2013).

Vitamin D deficiency may contribute to ASD pathology through multiple mechanisms. As a neurosteroid hormone, vitamin D plays critical roles in neurodevelopment, including neural cell proliferation (Siracusano et al., 2020), neurotransmitter balance (Fahim et al., 2025), oxidative stress responses (Pfeffer et al., 2018), and immune regulation (Prietl et al., 2013). Notably, vitamin D deficiency can lead to microglial hyperactivation, releasing IL-6 and TNF−α, which abnormally enhance synaptic phagocytosis and disrupt social behavior-related neural networks. Vitamin D deficiency also leads to abnormal neurodevelopment, neurotransmitter imbalance, reduced antioxidant capacity, and immune dysfunction, all consistent with core ASD pathologies (van Rooij et al., 2025). Furthermore, VDR gene polymorphisms are closely associated with ASD onset and behavioral manifestations; certain VDR genotypes correlate with symptom severity and behavioral features (e.g., hyperactivity) in children with ASD, suggesting that genetic interactions with vitamin D signaling may influence ASD pathogenesis (De Marzio et al., 2024).

Clinical intervention studies suggest that vitamin D supplementation may improve symptoms in children with ASD. Multiple RCTs and meta-analyses indicate that vitamin D supplementation effectively reduces scores on the Social Responsiveness Scale (SRS) and Childhood Autism Rating Scale (CARS), alleviating core symptoms (Saechua et al., 2024). However, results vary, with some studies failing to observe significant efficacy, possibly due to late intervention initiation (missing critical developmental windows), lack of baseline vitamin D stratification, low doses (<2000 IU/d), or absence of behavioral co-intervention (Seminario et al., 2022). Vitamin D deficiency is also associated with inflammatory status in ASD children, and supplementation reduces inflammatory markers, suggesting neuroprotection through immune-inflammatory regulation (Bischoff-Ferrari et al., 2005; Tripkovic et al., 2012; Zhang et al., 2019). Overall, there is a significant association between vitamin D deficiency and ASD risk, and supplementation holds potential for symptom improvement, but larger, multicenter, long-term RCTs are needed to clarify mechanisms and clinical value (Yu et al., 2022).

### 3.2 Childhood epilepsy and vitamin D

Epilepsy is a chronic brain disorder caused by abnormal neuronal discharges; its pathogenesis is not fully understood. Multiple clinical studies find that serum 25(OH)D levels in children with epilepsy, both before and during anti-seizure medication, are generally lower than in healthy peers, with deficiency rates exceeding 40%, suggesting that epilepsy itself or its treatment may affect vitamin D metabolism (Bouillon, 2017; Trump and Aragon-Ching, 2018). Some studies indicate that anti-seizure drugs are not the sole cause, and the epileptic condition itself may be associated with vitamin D metabolic disturbance (Sinopoli et al., 2024).

Vitamin D may exert anti-epileptic potential by modulating neuronal excitability. It influences calcium channel function, regulating neuronal excitability and synaptic plasticity, potentially raising seizure threshold (Zhang et al., 2006). Additionally, its immunomodulatory and anti-inflammatory actions may indirectly improve epileptic pathology. A Mendelian randomization study showed a causal association between vitamin D levels and risk of certain epilepsy subtypes (e.g., childhood absence epilepsy), suggesting that supplementation may reduce seizure risk (Meshkini et al., 2022).

In clinical intervention, vitamin D supplementation is gaining attention as an adjunctive treatment. In children on ketogenic diets, maintaining serum 25(OH)D above 40 ng/mL reduces urinary calcium excretion and lowers the risk of renal complications, indicating positive effects on bone health and drug safety (van den Heuvel et al., 2024). Some case reports suggest that vitamin D supplementation may help control seizure frequency, but most studies are small and observational, lacking high-quality RCTs to confirm efficacy and safety (Chassoux et al., 2024). In conclusion, vitamin D deficiency is common in children with epilepsy; vitamin D may participate in epileptogenesis through neuronal excitability and immune regulation; adjunctive supplementation has potential, but further studies are needed to determine optimal dose, timing, and long-term safety (Grant et al., 2025).

### 3.3 Attention-Deficit/Hyperactivity disorder and vitamin D

Attention-deficit / hyperactivity disorder is a neurodevelopmental disorder characterized by hyperactivity, impulsivity, and inattention. Studies show that vitamin D status in children with ADHD is inversely correlated with symptom severity. Multiple studies report that serum 25(OH)D levels in children with ADHD are significantly lower than in healthy controls, with higher deficiency rates, and vitamin D levels negatively correlate with core symptom scores (inattention, hyperactivity/impulsivity) (Bacchetta et al., 2022). Maternal vitamin D deficiency during pregnancy is also associated with increased ADHD risk in offspring, suggesting an early influence of vitamin D in ADHD pathogenesis (Chien et al., 2024).

Vitamin D plays important roles in neurotransmitter regulation and cognitive function. VDR is widely distributed in brain regions governing attention and behavior; vitamin D regulates the synthesis and metabolism of dopamine, serotonin, and other neurotransmitters, affecting neural network development and function. Vitamin D also participates in anti-inflammatory and antioxidant mechanisms, potentially preserving brain homeostasis by improving neuroinflammation and oxidative stress (Wimalawansa, 2019). Vitamin D supplementation shows potential for improving behavior and cognition in ADHD. RCTs indicate that vitamin D alone or combined with magnesium significantly improves behavior, attention, and social function in children with ADHD (Capozzi et al., 2020; Uwitonze and Razzaque, 2018). Several systematic reviews and meta-analyses suggest that vitamin D as an adjunct to methylphenidate reduces ADHD symptom scores with mild adverse effects. However, some studies observed no significant effect on inflammatory markers, indicating that mechanisms require further exploration (Khazai et al., 2008). In summary, vitamin D deficiency correlates with ADHD symptom severity; vitamin D is involved in ADHD pathogenesis through neurotransmitter regulation, cognitive function, and immune-inflammation; supplementation shows promise for improving behavior and cognition, but more high-quality studies are needed to determine optimal dosage and durability (Harrison, 1981; Macintyre et al., 1977; Pop et al., 2022). For comparison, we summarize the epidemiological evidence, core mechanistic pathways, clinical effects, and research gaps for these three disorders in Table 1.

**TABLE 1:** Summary of associations between vitamin D deficiency and pediatric neurological disorders.

Disorder	Epidemiological evidence	Core mechanistic pathways	Clinical efficacy	Key research gaps
Autism spectrum disorder	Serum 25-(OH)-D levels significantly lower in patients vs. controls (mean difference: –8 to –12 ng/mL); gestational vitamin D deficiency increases offspring ASD risk (OR = 1.5–2.0)	Microglial hyperactivation → complement C1q/C3-mediated synaptic over-pruning; impaired GABAergic interneuron maturation; reduced BDNF signaling	Supplementation (2000–4000 IU/day, 4–6 months) reduces CARS/SRS scores (SMD = –0.4 to –0.7); high heterogeneity (I^2^ > 60%)	Lack of birth cohort studies; no VDR genotype-stratified RCTs; limited evidence for optimal intervention window (<2 years)
Epilepsy	Vitamin D deficiency rate >40% (independent of AED use); Mendelian randomization confirms causal link between vitamin D status and childhood absence epilepsy risk	Modulated calcium channel function → reduced neuronal excitability; aberrant microglia-mediated synaptic pruning (complement-dependent); anti-inflammatory (reduced IL-6)	Supplementation (1000–4000 IU/day) reduces seizure frequency in some patients (small non-RCTs); unclear efficacy in drug-resistant epilepsy	No placebo-controlled RCTs; lack of subtype stratification; limited long-term safety data
Attention-deficit/hyperactivity disorder	Serum 25-(OH)-D levels inversely correlated with symptom scores (*r* = –0.3 to –0.5); gestational deficiency increases offspring ADHD risk (HR = 1.3–1.6)	Dysregulated dopaminergic signaling (reduced tyrosine hydroxylase expression); delayed prefrontal development; impaired oxidative stress/Nrf2 signaling	Vitamin D + magnesium or adjuvant to methylphenidate improves behavioral scores (SMD = –0.3 to –0.5); limited efficacy of monotherapy	Lack of long-term follow-up (>6 months); limited data on combined interventions (magnesium, omega-3); no assessment of executive function endpoints

## 4 Clinical application of vitamin D in pediatric neurological disorders: heterogeneity, challenges, and precision intervention

### 4.1 Current practice of vitamin D supplementation

Currently, the dosage, safety, and efficacy evaluation standards for vitamin D supplementation vary considerably, mainly influenced by disease type, patient age, and individual differences. Global pediatric supplementation doses range from 400 to 10,000 IU/d, with neurodevelopmental disorder patients typically receiving 2000–4000 IU/d, good tolerability, and an adverse event rate <5% (hypercalcemia rare) (Alnafisah et al., 2024). In extremely preterm infants, most US NICUs use 400 IU/d, initiating after a certain feeding volume is achieved, although some suggest exploring higher doses (e.g., 800 IU/d) in trials to optimize efficacy (Allen et al., 2015). In UK general practice, prescribed vitamin D doses and regimens also vary widely, with some not individually adjusted based on serum 25(OH)D levels (Itkonen et al., 2018).

Clinical trial results for different neurological diseases show diversity. In children with chronic neurological diseases, vitamin D deficiency is common, and supplementation is important for preventing osteoporosis and improving bone health, but direct evidence for neurological symptom improvement remains insufficient (Mpandzou et al., 2016). Children with ASD generally have lower vitamin D levels than controls, and deficiency may be a risk factor, but the efficacy of supplementation requires further validation (Kumar et al., 2022). In pediatric epilepsy, vitamin D levels correlate with executive function; low levels may worsen cognitive impairment, but no significant effect on quality of life in drug-resistant epilepsy has been shown (Hayden et al., 2021; Montero-Odasso et al., 2023). Additionally, the relationship between vitamin D and pediatric migraine has been noted; supplementation may reduce attack frequency and duration, improving quality of life (Benedetti et al., 2018).

Controversies in supplementation therapy mainly revolve around dosage standards, efficacy assessment, and the need for individualization. Some studies suggest that optimal vitamin D doses should be based on serum 25(OH)D levels, age, disease type, and nutritional status (Bischoff-Ferrari et al., 2020). In autoimmune neurological diseases like multiple sclerosis, the efficacy and safety of high-dose vitamin D remain debated (Manson et al., 2019; Zhu et al., 2025). The need for individualized therapy is increasingly recognized, as standardized regimens cannot meet the needs of all patients, especially those with genetic polymorphisms, metabolic differences, and different developmental stages (Algarin et al., 2024).

### 4.2 Risks of excessive vitamin D supplementation and neurotoxicity

Excessive long-term vitamin D intake may induce systemic toxicity and secondary neurological adverse reactions in children, which deserves full clinical attention alongside supplementation benefits. Excessive vitamin D leads to persistent hypercalcemia and hypercalciuria, the core toxic phenotypes of overdose (Rodrigues et al., 2022); corresponding neurological adverse manifestations in children include persistent irritability, sleep fragmentation (Chiloiro et al., 2024; Holmes and Kummerow, 1983), attention decline, and elevated seizure susceptibility, which may aggravate core symptoms of ASD, epilepsy and ADHD (Bouderlique et al., 2022). Existing pediatric toxicology data indicate that sustained serum 25(OH)D concentrations exceeding 100 ng/mL significantly increase neurological adverse risk, while routine therapeutic doses (2000–4000 IU/d) rarely reach this toxic threshold under regular monitoring (Alnafisah et al., 2024). Key clinical monitoring indicators include serum 25(OH)D, serum total calcium, and 24-h urinary calcium excretion (Yuan et al., 2025); pediatric patients with neurodevelopmental disorders require monthly routine biochemical monitoring during high-dose supplementation. Multiple risk modifiers regulate overdose susceptibility, including VDR genotype, concurrent calcium/magnesium supplementary intake, and baseline renal filtration function (Bouderlique et al., 2022). Clinicians should formulate dynamic dosage adjustment schemes based on regular biochemical monitoring to balance therapeutic efficacy and neurological safety.

### 4.3 Sources of heterogeneity in clinical trials

The inconsistency among current clinical trial results can be attributed to four main sources (Table 2): (1) Dosage and timing: Most trials used fixed doses (e.g., 400–2000 IU/d) without stratification by baseline serum 25(OH)D; intervention initiation ages varied widely (from neonatal to adolescence), potentially missing critical developmental windows (Jiao et al., 2022). (2) Outcome assessment tools: For example, in ASD studies, some used CARS, others SRS, which have different sensitivities for core symptoms (Gaengler et al., 2024). (3) Genetic background: VDR gene polymorphisms (e.g., FokI, TaqI) significantly affect responses to vitamin D supplementation, but very few studies have performed genotyping stratification (Malhotra and Lakade, 2025). (4) Neglect of co-interventions: Vitamin D metabolism and function depend on magnesium, calcium, vitamin K2, and other nutrients; many trials did not control or record intake of these factors, potentially masking true efficacy (Patrick and Ames, 2015).

**TABLE 2:** Sources of heterogeneity in clinical trials and standardized recommendations.

Heterogeneity source	Specific issues	Recommended solutions
Dosage and timing	• Fixed doses without baseline 25-(OH)-D stratification Variable intervention ages miss critical windows (e.g., synaptic burst)	• Stratify RCTs by baseline 25-(OH)-D levels (<20, 20–30, ≥30 ng/mL) Analyze early (<2 years) vs. late intervention subgroups
Outcome assessment tools	• ASD: variable CARS/SRS use ADHD: variable parent/teacher/objective testing No defined MCID	• Adopt consensus core outcomes (ASD: SRS + ADOS; ADHD: Conners + CPT) Standardize MCID reporting (e.g., ≥6-point SRS reduction = efficacy)
Genetic background	• VDR polymorphisms alter vitamin D signaling Trials rarely collect/analyze genotypes	• Genotype all participants for VDR polymorphisms Design trials with genotype stratification or enrichment
Concurrent interventions	• Dependence on magnesium, calcium, vitamin K2 Uncontrolled cofactor intake	• Standardize/record magnesium/calcium intake Use 2 × 2 factorial designs (vitamin D × magnesium) to test synergy

### 4.4 Challenges and future paths for precision intervention

On one hand, although vitamin D plays an important role in child neurodevelopment, standardization of vitamin D assays remains a major challenge. Differences between laboratories and methods affect accurate assessment of 25(OH)D levels, impacting clinical decision-making and comparability of research results. Internationally recognized standardized assays should be adopted in clinical practice and research to ensure data comparability and reliability (Macpherson et al., 2022).

On the other hand, precise assessment of vitamin D requirements at different child developmental stages lacks sufficient data. Children have diverse growth and development phases, with dynamic changes in vitamin D metabolism and requirements; uniform supplementation regimens cannot meet the needs of all stages, especially for extremely preterm infants, neurodevelopmental disorder patients, and children with chronic diseases. Future research should develop stage-specific vitamin D requirement models to guide individualized supplementation (Martineau et al., 2019).

**FIGURE 2:**
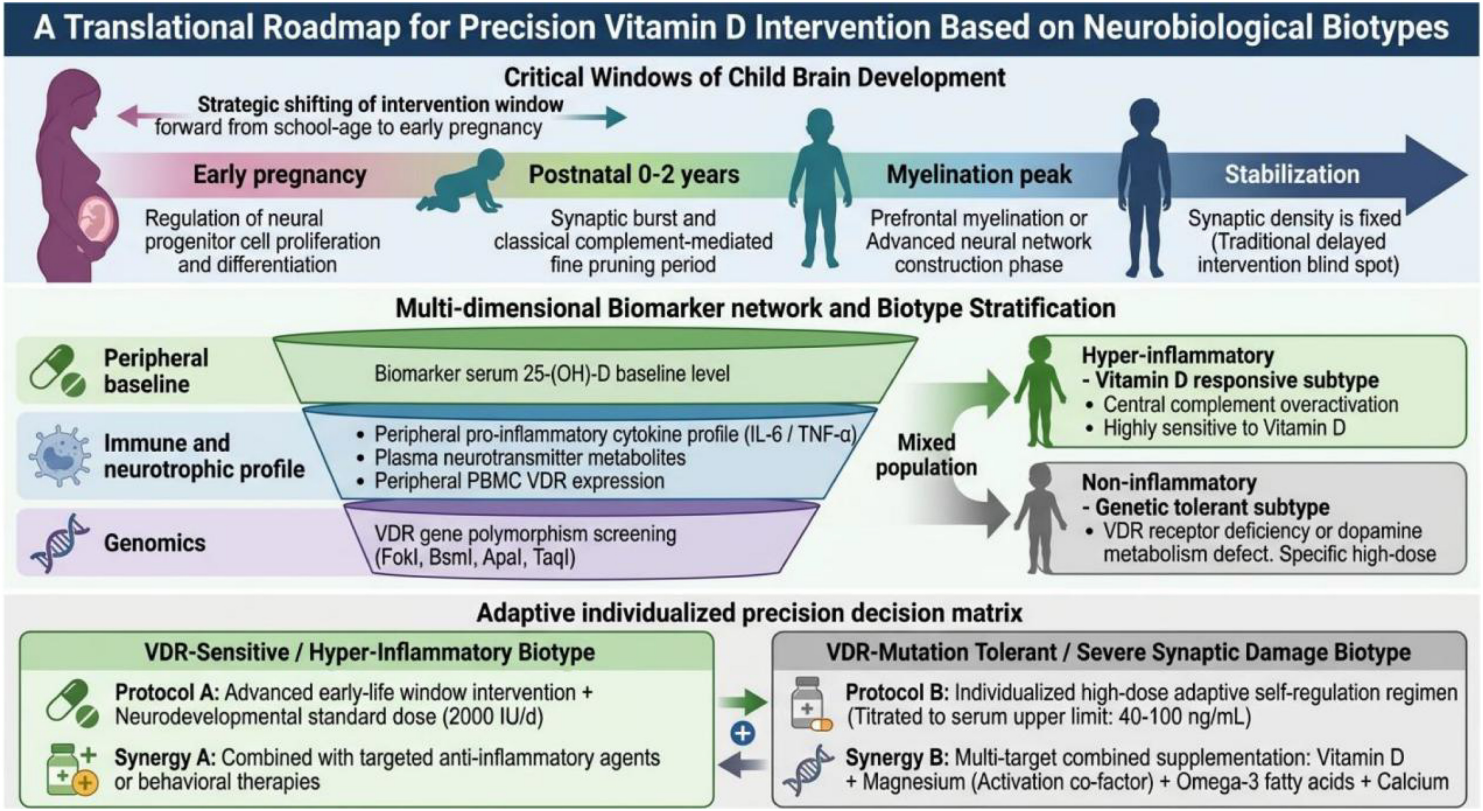
A translational roadmap for precision vitamin D intervention based on neurobiological biotypes.

To promote translational research on vitamin D in neurological diseases, multidisciplinary collaboration is needed, following these precision intervention pathways: ➀Developmental stage-based stratification: establish dynamic vitamin D requirement curves from pregnancy through neonatal, infant, and school-age periods, rather than a one-size-fits-all intake recommendation. ➁Biomarker-guided supplementation: in addition to serum 25(OH)D, explore combined use of inflammatory markers (IL-6, TNF−α), neurotransmitter metabolites, and VDR expression levels as predictors of efficacy. ➂Co-intervention trial design: standardize magnesium and calcium intake while supplementing vitamin D, and conduct RCTs of vitamin D combined with omega-3 or N-acetylcysteine. ➃VDR genotype-based adaptive design: prescreen for VDR polymorphisms, stratify children into genotype groups, and evaluate supplementation effects separately to achieve true individualization (Figure 2).

Furthermore, enhancing healthcare professionals’ awareness of the importance of vitamin D and its clinical application, and promoting the development of unified, standardized guidelines, will help improve comprehensive management of children with neurological disorders (Martineau et al., 2017).

## 5 Conclusion

Vitamin D, an essential fat-soluble vitamin, has received increasing attention for its role in child neurodevelopment and related disorders. This systematic review demonstrates that vitamin D exerts multiple functions through the “neuroimmune–synaptic plasticity axis” in neuroprotection, immune regulation, and synaptic function regulation. Cumulative preclinical and epidemiological evidence supports consistent correlations between vitamin D insufficiency and elevated risk of neurological disorders such as ASD, epilepsy, and ADHD, yet definitive causal relationships have not been fully established by existing human research data (Holló et al., 2014). However, existing clinical studies exhibit high heterogeneity, mainly due to inconsistency in dosage and timing, differences in outcome assessment tools, neglect of VDR genetic background, and lack of co-intervention control (Cheng et al., 2024; Guo et al., 2025; Miratashi Yazdi et al., 2017).

Therefore, future research should focus on the developmental stage-specific mechanisms of vitamin D, individual genetic differences, and the integration of basic and clinical research to define optimal supplementation regimens. Specifically, we propose the following research directions: 1. Conduct multicenter RCTs based on VDR genotyping to clarify “for whom it works”; 2. Establish dynamic models of vitamin D requirements at different child developmental stages to clarify “when to supplement”; 3. Explore synergistic effects of vitamin D with magnesium, omega-3, and other nutrients; 4. Develop disease-specific biomarkers of vitamin D responsiveness to promote the transition from empirical supplementation to precision medicine.

In summary, vitamin D, as a core regulator of the neuroimmune–synaptic plasticity axis, offers a new perspective and an actionable path for precision prevention and treatment of child neurodevelopmental disorders. Through multidisciplinary collaboration and precision medicine designs, it is expected to overcome current clinical bottlenecks and significantly improve long-term outcomes for children with neurodevelopmental disorders.
